# Supplementary material for: Predation and fragmentation portrayed in the statistical structure of prey time series
Source: BMC Ecol. 2009 May 6;9:10. doi: 10.1186/1472-6785-9-10 (PMC2689204; doi:10.1186/1472-6785-9-10)
Supplement: Additional file 2 — Voles and related classes ODDox Documentation. ODDox documentation of the agent-based model (ALMaSS) applied by Hendrichsen et al. The documentation is started by activating main.html. [file 1472-6785-9-10-S2.zip › Vole_ODDox/_winter_wheat_8cpp.html]

ALMaSS ODDox: WinterWheat.cpp File Reference

- Main Page
- Related Pages
- Classes
- Files

# WinterWheat.cpp File Reference

---

## Detailed Description

**WinterWheat.cpp This file contains the source for the WinterWheat class**   

by Chris J. Topping   
Version of June 2003   
All rights reserved.   
  
With additions as noted in:   
Doxygen formatted comments in July 2008   

`#include "ls.h"`  
`#include "WinterWheat.h"`  

|  |  |
| --- | --- |
|  |  |
| Variables | |
| CfgFloat | cfg\_fungi\_app\_prop |
| CfgFloat | cfg\_greg\_app\_prop |
| CfgFloat | cfg\_herbi\_app\_prop |
| CfgFloat | cfg\_ins\_app\_prop |

---

## Variable Documentation

CfgFloat cfg\_fungi\_app\_prop

Referenced by WinterWheat::Do().

CfgFloat cfg\_greg\_app\_prop

Referenced by WinterWheat::Do().

CfgFloat cfg\_herbi\_app\_prop

Referenced by WinterWheat::Do().

CfgFloat cfg\_ins\_app\_prop

Referenced by WinterWheat::Do().

---

Generated on Thu Jan 22 14:13:45 2009 for ALMaSS ODDox by 
 1.5.6
